# Recurrent Ischemic Stroke and Transient Ischemic Attack: Risk of Single and Multiple Recurrence

**DOI:** 10.3390/jcm13195744

**Published:** 2024-09-26

**Authors:** Moon-Ho Park, Sang-Hun Lee, Jin-Man Jung

**Affiliations:** Department of Neurology, Korea University Ansan Hospital, Ansan 15355, Republic of Korea; huny0029@naver.com (S.-H.L.); sodium75@hanmail.net (J.-M.J.)

**Keywords:** ischemic stroke, transient ischemic attack, recurrence, multiple, single, risk

## Abstract

**Background/Objectives**: Efforts have been made toward primary or secondary stroke or transient ischemic attack (TIA) prevention. However, little attention has been paid to recurrent stroke or TIA. This study investigated risk factors for multiple or single recurrent stroke or TIA. **Methods**: Data from 3646 patients with ischemic stroke or TIA were obtained from the Korea University Ansan Hospital Stroke Center between March 2014 and December 2021, using the prospective institutional database of the Korea University Stroke Registry. The associations between clinical features and recurrent stroke or TIA were assessed using bivariable and multivariable Cox models. **Results**: Recurrent stroke or TIA was associated with male sex (adjusted hazard ratio (HR) 1.95, 95% confidence interval (CI) 1.42–2.80), hypertension (HR 1.49, 95% CI 1.00–2.23), diabetes mellitus (HR 1.54, 95% CI 1.13–2.13), an etiologic subtype of transient ischemic attack (HR 1.88, 95% CI 1.09–3.16), white matter changes (HR 1.62, 95% CI 1.05–2.38), and cerebral microbleeds (HR 1.79, 95% CI 1.26–2.59). Multiple recurrent stroke or TIA was associated with male sex (HR 3.86, 95% CI 1.94–11.55), diabetes mellitus (HR 2.40, 95% CI 1.31–4.53), and anemia (HR 4,58, 95% CI 2.31–10.44). **Conclusions**: Given the risk factor profiles for recurrent stroke or TIA, risks differed among patient subgroups and were based on multiple or single recurrences. It may exert an effect as a prognostic indicator in the high risk of recurrences.

## 1. Introduction

Cerebrovascular disease (CVD) includes conditions, such as stroke and transient ischemic attack (TIA), that remain major public health concerns. Proper evaluation including a comprehensive and timely evidence-based workup could aid in the prevention of CVD [1,2].

While the rates of CVD have decreased in recent years, many survivors experience recurrent attacks [1,3,4]. Although there are primary or secondary prevention guidelines for CVD [1,2], recurrent attacks can, at least to some extent, be considered a failure of prevention guidelines. In recurrent attacks, neurological damage is usually severe and challenging to treat and such cases are associated with higher disability and mortality rates and higher hospitalization costs compared with first-ever attack cases [5,6]. Unfortunately, little attention has been paid to multiple recurrent attacks. It has not been well known to accurately discriminate or separate patients with CVD at high and low risk of multiple recurrences. To implement effective prevention strategies in a healthcare system, the risks of individual multiple recurrent attacks following ischemic stroke and TIA need to be elucidated.

In this study with CVD, we investigated the clinical characteristics of recurrent attacks and the risk factors of multiple or single recurrent attacks compared to those of first-ever attacks.

## 2. Materials and Methods

### 2.1. Participants

We analyzed the anonymized data from the Korea University Stroke Registry. The design of the database has been previously described [7]. Briefly, we assessed consecutive patients with CVD in August 2023, who were admitted to the Stroke Center of Korea University Ansan Hospital between March 2014 and December 2021. De-identified information on demographics, clinical evaluation, neurological examination, and the characteristics of CVD were obtained.

**Figure 1:**
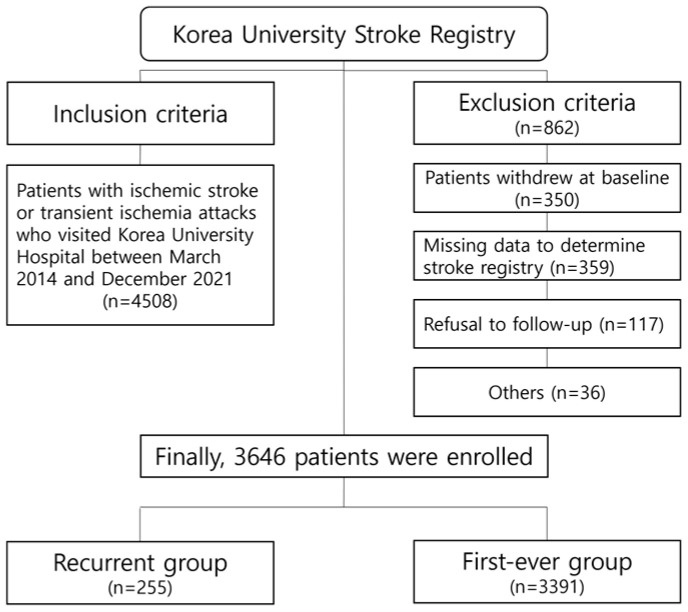
Flowchart of patient inclusion and exclusion.

During the study period, 4508 patients were enrolled in the cohort. In total, 862 were excluded from the final analysis according to prespecified criteria (Figure 1). The final analysis included 3646 patients, including 255 (7.0%) who experienced recurrence and 3391 who had a first-ever attack.

Patients were included if they had an ischemic stroke or TIA documented on the discharge. Patients with intracranial hemorrhage, subarachnoid hemorrhage, or subdural hematoma identified by computed tomography (CT) scan or magnetic resonance imaging were not included. Patients were excluded if they were inadequate for analysis, such as withdrawal, missing, or refusal to follow-up. Stroke was defined as per the World Health Organization [8] as rapidly developing clinical signs of focal (or global) disturbance of cerebral function, with symptoms lasting 24 h or longer or leading to death, with no apparent cause other than vascular origin. Ischemic stroke was defined as stroke consistent with focal brain ischemia and imaging confirmation of acute vascular ischemic pathology. TIA was defined as the loss of cerebral or ocular function for less than 24 h, presumably owing to atherosclerotic causes, and without evidence of acute infarction [9]. The definition of recurrence was the same as that of the first-time CVD, which was defined as a new acute neurologic deficit that is vascular in etiology or worsening of a former deficit not attributed to brain shift, edema, hemorrhagic transformation, concurrent illness, hypoxia, or drug toxicity, with neuroimaging consistent with recent infarction or TIA, occurring at least 24 h after the onset of the index event [10]. CVD occurring in patients who had a definite TIA, but had a subsequent CVD within 24 h of onset of TIA, were also excluded from recurrence. All patients were followed for 3 and 12 months after each event or until recurrent attack. At 3 months, clinical outcome was assessed using the modified Rankin Scale. At 12 months, recurrence or other clinical problems were checked. If the patient was seen in the stroke clinic, 3- and 12-month evaluations were obtained primarily by the stroke neurologists in charge of the patient. If the patient was not seen, research nurses obtained 12-month information by a structured telephone interview.

Standard systemic investigations were performed for every patient, including routine laboratory tests, chest radiography, 12-lead electrocardiography, and brain images. Routine laboratory tests included a complete blood count, electrolytes, glucose, renal function, liver function, lipid profile, and homocysteine levels. Blood samples were obtained from all participants after at least 8 h of fasting in the morning after admission for laboratory evaluation. Transcranial Doppler, carotid duplex sonography, transthoracic echocardiography, transesophageal echocardiography, and 24 h Holter electrocardiography monitoring were performed in selected patients. Brain CT and/or magnetic resonance imaging were performed. Each patient underwent at least one vascular imaging scan, including conventional angiography, arterial imaging using magnetic resonance angiography, CT angiography, or duplex ultrasound imaging. 

This study was approved by the Institutional Review Board of Korea University Ansan Hospital (approval no. 2021AS0213).

### 2.2. Clinical Assessment

Age at admission was categorized into three groups (<60, 60–74, and ≥75 years). Body mass index (BMI) was calculated as weight in kilograms divided by height in meters squared (kg/m^2^) and categorized into four groups (underweight, <18.5; normal weight, 18.5–22.9; overweight, 23.0–24.9; obese, ≥25.0). The weight and height required for BMI measurement were measured within 24 h of admission. Hypertension was defined as a systolic blood pressure of at least 140 mmHg and a diastolic blood pressure of at least 90 mmHg, or as a previous diagnosis or prescription for antihypertensive medication. Diabetes mellitus was defined as a fasting serum glucose level ≥126 mg/dL, a non-fasting serum glucose level ≥200 mg/dL, a hemoglobin A1c level ≥6.5%, or a history of insulin therapy and/or oral hypoglycemic drugs. Atrial fibrillation was defined as persistent atrial arrhythmia, with irregular R-R intervals and no clear repetitive P waves, and was diagnosed with an electrocardiogram, 24 h Holter, or continuous electrocardiogram monitoring during hospitalization. Dyslipidemia was defined as a total cholesterol level ≥200 mg/dL. 

The type of ischemic stroke was classified according to the Trial of Org 10,172 in the Acute Stroke Treatment (TOAST) classification system [11]. The subtypes were classified into five categories based on etiology using the TOAST classification: large artery disease (large artery atherosclerosis, ≥50% stenosis), cardioembolism (several causes of cardioembolic disease, including atrial fibrillation, myocardial infarction with left ventricular thrombus, and infective or inflammatory endocarditis), small-vessel occlusion (lacunes), a stroke of other determined etiology (uncommon stroke causes, such as nonatherosclerotic vasculopathies, hypercoagulable states, or hematologic disorders), and a stroke of undetermined etiology (two or more etiology, negative, or incomplete examination). The extent of the diagnostic workup and subtypes of ischemic stroke and TIA were determined primarily by the stroke neurologists in charge of the patients. CVD subtypes were confirmed at a monthly stroke registry meeting. 

CVD is associated with abnormal clinical features, including anemia [12], leukocytosis [13], elevated high-sensitivity C-reactive protein (CRP) levels [13], hyperhomocysteinemia [14], reduced kidney function [15], white matter changes [16], and cerebral microbleeds (CMBs) [17]. In this study, the cut-off points of the clinical features were used to define and categorize abnormal levels: anemia was defined as hemoglobin <12 g/dL in females and <13 g/dL in males; leukocytosis was defined as a white blood count >12,000/μL; hyperhomocysteinemia ≥15 μmol/L; elevated CRP 0.3–1.0 or ≥1.0 mg/L; reduced kidney function as decreased estimated glomerular filtration rate (eGFR) according to the Chronic Kidney Disease Epidemiology Collaboration formula (estimated glomerular filtration rate <60 mL/min per 1.73 m^2^); advanced white matter changes were defined using the visual rating scale proposed [16]; and CMBs were small (<10 mm), hypointense, and ovoid or rounded regions on T2-weighted gradient-recalled echo or susceptibility-weighted imaging. The severity of neurological deficits at admission was rated using the National Institutes of Health Stroke Scale (NIHSS) [18]; poor initial NIHSS was defined as an NIHSS score ≥5. Thrombolysis was categorized as intravenous thrombolysis or intra-arterial thrombectomy.

### 2.3. Statistical Analysis

All participants were categorized into two groups: the recurrent group, which included patients with recurrent ischemic stroke or TIA, and the first-ever group, which included patients with first-ever ischemic stroke or TIA. The recurrent group was sub-categorized into single recurrent attack and multiple recurrent attack (2 or more than recurrent attacks) groups. Categorical variables were described in terms of frequencies and percentages. Differences in characteristics among groups were analyzed using Pearson’s chi-square (χ^2^) test or Fisher’s exact test, as applicable. The pairwise z-test with Bonferroni correction was used to determine the significance of the contribution for each subgroup of variables.

Kaplan–Meier curves were plotted and compared using log-rank tests. To identify potential predictors of recurrent attack, we selected the variables with *p* < 0.2 in a bivariate analysis. These potential variables of recurrent attack were used to generate a predictive model on each recurrent subgroup by multivariable Cox regression models using bootstrapping methods with a significant association at *p* < 0.05 in comparison with the first-ever group. The Cox-proportional hazard model assumption was assessed graphically and using a global test based on Schoenfeld residuals at the level of *p* > 0.05. The linear relationship between residual and time was not significant, showing that recurrent attacks did not depend on time changes. The Cox regression models were used to calculate hazard ratios (HRs) and 95% confidence intervals (CIs). Statistical significance was indicated when the two-tailed *p* value was <0.05. Statistical analyses were performed using R version 4.2.1 software (R Foundation for Statistical Computing, Vienna, Austria) and SPSS version 20.0 (IBM SPSS, Chicago, IL, USA).

## 3. Results

The clinical characteristics of the participants in the two groups are presented in Table 1. The recurrent group had significantly different proportions of sex, BMI, hypertension, diabetes mellitus, TOAST classification, anemia, CRP, decreased eGFR, white matter changes, and CMBs compared with the first-ever group.

**Figure 2:**
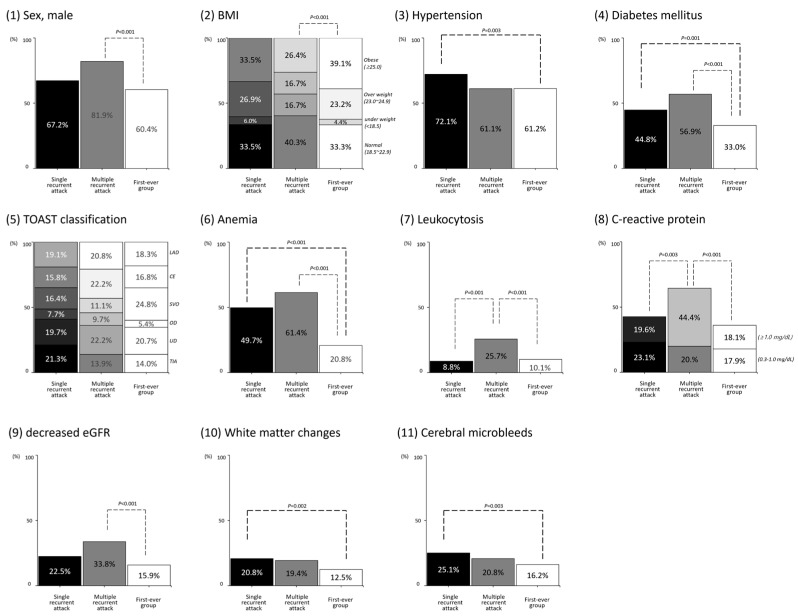
Difference in clinical characteristics among single recurrent attack, multiple recurrent attack, and first-ever groups. *p* values were calculated from the chi-square test with a Bonferroni correction to determine the significant distribution among single recurrent attack, multiple recurrent attack, and first-ever groups. *p* values and black dotted lines indicate statistical significance (*p* value <0.05/3).

In the recurrent group, there were different distributions of clinical characteristics in the single recurrent attack and multiple recurrent attack subgroups (Table 1, Figure 2). In the single recurrent attack, there were significant differences in hypertension, diabetes mellitus, anemia, leukocytosis, CRP, white matter changes, and CMBs compared with the first-ever group or multiple recurrent attack group. In the multiple recurrent attack group, there were significant differences in the distributions of sex, BMI, diabetes mellitus, anemia, leukocytosis, CRP, and decreased eGFR compared with the first-ever group or single recurrent attack group.

**Figure 3:**
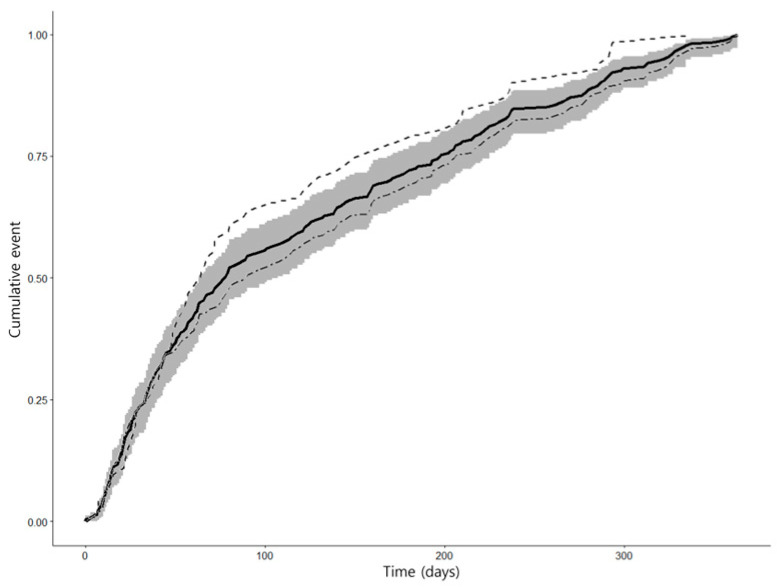
Kaplan–Meier curve for the recurrent group. The cumulative event curve for recurrence is indicated by the black solid line with 95% CI (gray area). The curve for single recurrent attacks is indicated by a gray double-dashed line, and the curve for multiple recurrent attacks is indicated by a gray single-dashed line.

In the 255 patients in the recurrent group, the total follow-up until the recurrent event was 30,463 patient days. The Kaplan–Meier estimate for an interval of recurrence after the index attack was 119.46 days (95% CI 106.85–132.07) (Figure 3). Upon stratification of the subgroups by recurrent stroke, the interval of recurrence after the index attack was 126.45 days (95% CI 110.96–141.94) in the single recurrent attack and 101.71 days (95% CI 81.02–122.40) in the multiple recurrent attack. The log-rank test for the equality between interval distributions showed no significant difference (χ^2^ statistic 3.693, *p* = 0.055) between single recurrent attacks and multiple recurrent attacks.

**Figure 4:**
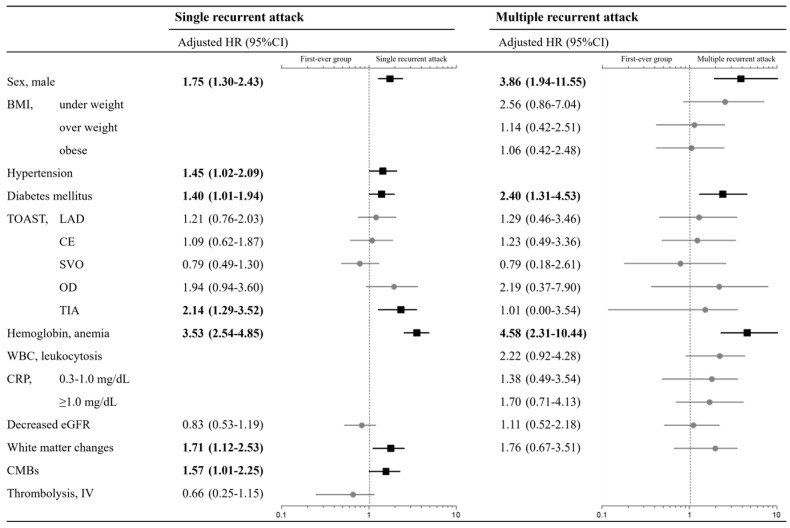
Multivariable Cox regression model for single recurrent attack or multiple recurrent attack. Data are expressed as hazard ratios with 95% confidence intervals in parentheses. Values in bold font and black squares with black lines indicate statistical significance (*p* < 0.05). Values in normal font and gray circles with gray lines indicate no statistical significance.

In the Cox regression analysis, the final multivariable analysis revealed that male sex, BMI, hypertension, diabetes mellitus, TOAST classification, anemia, leukocytosis, CRP, decreased eGFR, white matter changes, and CMBs were associated with the recurrent group (Table 2). In the subgroup of recurrence, single recurrent attacks were associated with the male sex, hypertension, diabetes mellitus, TOAST classification, anemia, white matter changes, and CMBs; multiple recurrent attacks were associated with the male sex, diabetes mellitus, and anemia (Figure 4).

We then included time-dependent effects for these clinical characteristics and time change points of effects were chosen based on the plot of Schoenfeld residuals. Since Schoenfeld residuals reveal the functional form of the time-dependent effect, if the tendency line of the plot is close to a horizontal line, there is evidence that a usual Cox model is appropriate.

In the residual assessment of recurrence, the fitting curve of the scatter plot was close to a horizontal line. In addition, all *p* values of the test were greater than 0.05, proving that recurrence and time were independent of each other and indicating that the prediction ability of the recurrent stroke was less affected by time; thus, there is evidence that a usual Cox model is appropriate.

## 4. Discussion

This study investigated the risk profile of patients with recurrent stroke or TIA compared with patients with a first-ever stroke or TIA. The distinctive feature of this study is that we investigated not only risk factors of the recurrent group but also distinguished between single recurrent attacks and multiple recurrent attacks. We observed a different risk profile between the subgroups of recurrence. Male sex, hypertension, diabetes mellitus, TIA, anemia, white matter changes, and CMBs were identified as risk factors for single recurrent attacks, while male sex, diabetes mellitus, and anemia acted as risk factors for multiple recurrent attacks. 

This study reported that the cumulative risk of recurrence was 7.0% at 1 year. While direct comparison may be contentious, this rate is lower than that of a meta-analysis that estimated a pooled cumulative recurrent risk of 11.1% at 1 year [19]. Despite the subgroup inclusion of TIA, this risk difference could be the declined prevalence of recurrence with decreased trends in CVD [4] and optimal use of secondary prevention strategies [2].

To the best of our knowledge, this is the first study that evaluated the risk factors of recurrent CVD including not only single recurrent attacks but also multiple recurrent attacks. The recurrence of CVD is frequent [1,3,4] and is devastating [5,6]. A previous study estimated that at least 80% of recurrent vascular events after CVD might be prevented with the use of a comprehensive approach [20]. Patients with CVD should be provided with opportunities to improve their knowledge of their risk conditions, which are important in the specific recommendations for the prevention of further recurrent attacks.

This study showed that male sex, diabetes mellitus, and anemia were common risk factors for both subgroups of recurrence. In addition, hypertension, TIA, white matter changes, and CMBs were risk factors for single recurrent attacks. Although no definite factor has been consistently associated with the recurrence of CVD, a previous meta-analysis reported that recurrence was related to hypertension, diabetes mellitus, atrial fibrillation, TIA, and high stroke severity [21].

This study reported that male sex was associated with single recurrent attacks and multiple recurrent attacks. While the causes of the sex differences in recurrence have yet to be fully elucidated, a higher recurrence risk in men compared with women has been previously reported [22]. Differences may be explained by differences in sex steroid hormones, life expectancy, or age at the time of CVD onset [22,23]. Future studies need to further investigate the sex differences in recurrence.

Diabetes mellitus is an important risk factor for CVD [1,2,24]. All patients with CVD were recommended screening for diabetes and glycemic control. Diabetes mellitus shares multiple aspects with CVD including micro-angiopathy or metabolic abnormalities that might contribute to vascular dysfunction [24,25]. Previous studies have reported that the recurrence is related to diabetes mellitus [25,26]. The present study reported that diabetes mellitus was related to single recurrent attacks and multiple recurrent attacks. 

Hematologic disorders have been associated with CVD [12]. In addition to hematologic disorders such as polycythemia vera, sickle cell anemia, and essential thrombocythemia that cause CVD, anemia is also a risk factor associated with unfavorable outcomes in patients with CVD [12]. A previous study reported that the one-year rates of recurrence were significantly higher in patients with anemia [27]. The present study provides further support for the relationship between anemia and the recurrence of CVD. Although the precise mechanism remains unknown, it is conjectured that oxygen deprivation, microcirculatory dysfunction, inflammatory response, and exacerbation of small-vessel occlusive pathology in anemia may be contributing factors for recurrence [12,27]. 

This study reported that some risk factors such as hypertension, TIA, white matter changes, and CMBs were associated with single recurrent attacks. Hypertension is usually considered the most important risk factor for CVD, and it is an independent predictor of recurrence [1,21,28]. Treatment of hypertension or blood pressure reduction can reduce the risk of recurrence [1]. In the present study, hypertension was associated with single recurrent attacks.

The etiologic subtype of stroke may be associated with the recurrence of CVD [25,28]. This study included TOAST subtypes as well as TIA in analysis and found that TIA was associated with single recurrent attacks. Previous studies have reported inconsistent results that no differences in the prevalence of recurrence among TOAST subtypes [29,30] or specific subtypes such as large artery disease [25] or cardioembolism [31] were associated with recurrent attacks. Although it was suggested that recurrent rates of the subtypes may have depended on different pathogenesis which reflect instability in the stenotic arteries and in micro-emboli from the heart [32], it has not been firmly established. Another previous study reported that TIA, especially multiple TIAs, was associated with an increased subsequent risk of CVD [32]. Preventing a TIA and preventing a stroke are equally important, and the current American Heart Association and the American Stroke Association guidelines apply to both [1]. This subsequent risk of recurrence after TIA might be explained by embolisms or a hemodynamic mechanism [32]. The present study found that TIA was associated with single recurrent attacks but not multiple recurrent attacks, and further study is required.

White matter changes and CMBs are associated with risk factors for small-vessel disease [33,34], and they can increase the risk of recurrence [34,35]. The mechanisms of CVD with white matter changes or CMBs involve reduced white blood flow, vessel fragility, and endothelial instability, which might be explained in the same context as patients with lacunar infarction [36,37]. The present study found that white matter changes and CMBs were associated with single recurrent attacks.

Recurrent attacks are associated with increased disability and mortality rates compared with no recurrent CVD [5,6]. Thus, a proper workup and evaluation of risk factors are essential. Although this study cannot investigate the differences in pathophysiology between single recurrent attacks and multiple recurrent attacks, it differentiated the recurrent group and provided the risk factors associated with each subgroup of recurrence. Effective management of these factors can reduce the risk of recurrence. As such, it is necessary that patients be provided opportunities to improve their knowledge of their risk conditions and personalized methods to ensure maximal treatment to prevent recurrence [38].

The main contribution of this study is that different risk factors of single recurrent attacks and multiple recurrent attacks were analyzed after adjusting for relevant confounders in a multivariate analysis. A predefined registry protocol and the use of electronic medical records with imaging data provide precise and accurate clinical information in both the recurrent group and the first-ever recurrent group.

This study has several limitations. First, we may have underestimated the true incidence of recurrence, as recurrent events were identified in patients who had to be admitted to a hospital within our region. Second, our findings are from a Korean population, and the study results may not be applicable to patients with other demographics. The third limitation is the relatively short one-year follow-up to determine recurrence and some missing data. Fourth, in part due to the hypothesis-generating nature of the study, no formal sample size estimation was performed, and the strategy was just to collect the maximal number of informative cases. Fifth, antiplatelet or anticoagulant medications can affect the prevention of the recurrence of CVD. A more detailed evaluation using these medications and their effect should be performed in further study.

## 5. Conclusions

The associated increased risk of the recurrent group was particularly high in patients with male sex, high BMI, hypertension, diabetes mellitus, TOAST classification, anemia, leukocytosis, CRP, decreased eGFR, white matter changes, and CMBs. Multiple recurrence was associated with male sex, diabetes mellitus, and anemia. These risk factors should be evaluated in patients with CVD to identify those with the greatest risk of recurrence who might benefit from future studies focused on additional intervention in further prevention.

## Figures and Tables

**Table 1:** Clinical characteristics of the enrolled participants.

	Recurrent Group			First-Ever Group	
	Total (n = 255)	Single (n = 183)	Multiple (n = 72)	(n = 3391)	*p* Value
Demographics					
age, yrs					0.900
<60	86 (33.7%)	63 (34.4%)	23 (31.9%)	1167 (34.4%)	
60–74	91 (35.7%)	67 (36.6%)	24 (33.3%)	1178 (34.7%)	
≥75	78 (30.6%)	53 (29.0%)	25 (34.7%)	1046 (30.8%)	
^b^ sex, male	182 (71.4%)	123 (67.2%)	59 (81.9%)	2049 (60.4%)	<0.001
^b^ BMI, kg/m^2^					0.001
normal (18.5–22.9)	90 (35.4%)	61 (33.5%)	29 (40.3%)	1125 (33.3%)	
underweight (<18.5)	23 (9.1%)	11 (6.0%)	12 (16.7%)	147 (4.4%)	
overweight (23.0–24.9)	61 (24.0%)	49 (26.9%)	12 (16.7%)	784 (23.2%)	
obese (≥25.0)	80 (31.5%)	61 (33.5%)	19 (26.4%)	1321 (39.1%)	
currently smoking	60 (23.5%)	44 (24.0%)	16 (22.2%)	899 (26.5%)	0.306
Cardiovascular comorbidity					
^a^ hypertension	176 (69.0%)	132 (72.1%)	44 (61.1%)	2076 (61.2%)	0.010
^a,b^ diabetes mellitus	123 (48.2%)	82 (44.8%)	41 (56.9%)	1120 (33.0%)	<0.001
atrial fibrillation	51 (20.0%)	35 (19.1%)	16 (22.2%)	577 (17.0%)	0.200
dyslipidemia	118 (59.6%)	92 (60.9%)	26 (55.3%)	2102 (63.3%)	0.300
^a^ TOAST classification					0.003
LAD	50 (19.6%)	35 (19.1%)	15 (20.8%)	621 (18.3%)	
CE	45 (17.6%)	29 (15.8%)	16 (22.2%)	569 (16.8%)	
SVO	38 (14.9%)	30 (16.4%)	8 (11.1%)	840 (24.8%)	
OD	21 (8.2%)	14 (7.7%)	7 (9.7%)	183 (5.4%)	
UD	52 (20.4%)	36 (19.7%)	15 (22.2%)	702 (20.7%)	
TIA	49 (19.2%)	39 (21.3%)	10 (13.9%)	476 (14.0%)	
Clinical feature					
^a,b^ hemoglobin, anemia	133 (53.0%)	90 (49.7%)	43 (51.4%)	704 (20.8%)	<0.001
^b^ WBC, leukocytosis	34 (13.5%)	16 (8.8%)	18 (25.7%)	341 (10.1%)	0.080
^b^ CRP					0.003
0.3–1.0 mg/dL	42 (22.3%)	33 (23.1%)	9 (20.0%)	557 (17.9%)	
≥1.0 mg/dL	48 (25.5%)	28 (19.6%)	20 (44.4%)	565 (18.1%)	
hyperhomocysteinemia	32 (21.1%)	28 (23.5%)	4 (12.1%)	625 (20.0%)	0.700
^a,b^ decreased eGFR	65 (25.7%)	41 (22.5%)	24 (33.8%)	538 (15.9%)	<0.001
^a^ white matter changes	52 (20.4%)	38 (20.8%)	14 (19.4%)	425 (12.5%)	<0.001
^a^ CMBs	61 (23.9%)	46 (25.1%)	15 (20.8%)	548 (16.2%)	0.001
poor initial NIHSS	73 (30.3%)	48 (28.2%)	25 (35.2%)	1046 (31.8%)	0.600
Clinical management					
thrombolysis, IV	14 (5.5%)	9 (4.9%)	5 (6.9%)	289 (8.5%)	0.090
thrombectomy, IA	4 (1.6%)	1 (0.5%)	3 (4.2%)	91 (2.7%)	0.300

Data are expressed as number (percentage). Because of some missing variables, the number of available datapoints was 3631 for BMI, 3518 for dyslipidemia, 3635 for anemia, 3635 for leukocytosis, 3307 for CRP, 3282 for hyperhomocysteinemia, 3639 for eGFR, and 3530 for NIHSS; the number of datapoints for other variables was 3646. The *p* values were unadjusted in the log-rank test for comparison between the recurrent group and the first-ever group. For post hoc analysis with a comparison of three diagnostic groups (single recurrent attack, multiple recurrent attack, or first-ever group), the value of 0.05 divided by 3 was used to determine statistical significance (^a^ significant between single recurrent attack and first-ever group, ^b^ significant between multiple recurrent attack and first-ever group).

**Table 2:** Bivariable and multivariable Cox regression analyses of the recurrent group.

		Crude HR	(95% CI)	Adjusted HR	(95% CI)
Age ^a^	60–74 yrs	1.05	(0.78–1.41)		
	≥75 yrs	1.01	(0.74–1.37)		
Sex	male	1.60	(1.22–2.10)	**1.95**	(**1.42**–**2.80**)
BMI ^b^	underweight	1.89	(1.20–2.99)	1.44	(0.80–2.40)
	overweight	0.98	(0.71–1.35)	1.26	(0.87–1.89)
	obese	0.77	(0.57–1.03)	0.99	(0.70–1.44)
Currently smoking		0.86	(0.64–1.15)		
Hypertension		1.40	(1.07–1.82)	**1.49**	(**1.00**–**2.23**)
Diabetes mellitus		1.84	(1.44–2.35)	**1.54**	(**1.13**–**2.13**)
Atrial fibrillation		1.21	(0.89–1.64)		
Dyslipidemia		0.86	(0.65–1.15)		
TOAST ^c^	LAD	1.09	(0.74–1.61)	1.13	(0.70–1.81)
	CE	1.07	(0.72–1.59)	1.14	(0.71–1.78)
	SVO	0.62	(0.41–0.94)	0.72	(0.44–1.16)
	OD	1.54	(0.93–2.56)	1.88	(0.93–3.51)
	TIA	1.36	(0.92–2.01)	1.88	(1.09–3.16)
Hemoglobin, anemia		4.02	(3.14–5.15)	**3.64**	(**2.63**–**5.19**)
WBC, leukocytosis		1.39	(0.97–1.99)	1.10	(0.64–1.67)
CRP ^d^	0.3–1.0 mg/dL	1.52	(1.06–2.19)	1.31	(0.86–1.89)
	≥1.0 mg/dL	1.71	(1.21–2.42)	0.97	(0.63–1.47)
Hyperhomocyteinemia		1.07	(0.72–1.58)		
Decreased eGFR		1.78	(1.34–2.36)	0.82	(0.55–1.15)
White matter changes		1.75	(1.29–2.37)	**1.62**	(**1.05**–**2.38**)
CMBs		1.60	(1.20–2.13)	**1.79**	(**1.26**–**2.59**)
Poor initial NIHSS		0.94	(0.71–1.23)		
Thrombolysis, IV		0.63	(0.37–1.09)	0.74	(0.36–1.27)
Thrombectomy, IA		0.59	(0.22–1.59)		

Variables with *p* value less than 0.20 during bivariable analysis for crude hazard ratio were exported to multivariable analysis to examine the adjusted hazard ratio. Values in bold font indicate statistical significance (*p* < 0.05) in multivariable analysis. ^a^ The reference category was <60 years. ^b^ The reference category was normal weight (BMI 18.5–22.9 kg/m^2^). ^c^ The reference category was UD (stroke of undetermined etiology). ^d^ The reference category was <0.3 mg/dL.

## Data Availability

The raw data supporting the conclusions of this article will be made available by the authors upon request.
